# Supplementary material for: Molecular force gates regulate mechanical transmission across the nuclear envelope
Source: Nucleus. 2026 Nov 8;17(1):2756751. doi: 10.1080/19491034.2026.2756751 (PMC13757650; doi:10.1080/19491034.2026.2756751)
Supplement: Supplementary file 1 [file KNCL_A_2756751_SM0274.docx]

**Supplementary Figure 1**	7	,	-,	,	, 0 1 0	5

-	30	,	,	( ,

6	6	6	6	6	6

Figure S1. Force–time, force–extension, mean-force, and peak-force responses of single-point proline mutants compared with reference constructs. Two single-point proline substitutions were introduced to probe the mechanical roles of specific residues: ΔSS R6877P (red), in which arginine 6877 was replaced by proline in the disulfide-deleted (ΔSS) background, and M6875P (dark green), in which methionine 6875 was replaced by proline in the wild-type background. Reference constructs—WT (cyan), ΔSS (purple), KASH-2P (light green), and ΔSS-KASH-2P (orange)—are shown for comparison. (a) Force as a function of time. Force traces were smoothed using a centered 1-ns moving average. (b) Force as a function of extension. Force was binned by extension within each replicate before replicate averaging. In (a) and (b), solid lines represent the mean of three replicates and shaded regions represent ±1 SD. (c) Full-trajectory mean force. Each point represents the mean force from one 50-ns replicate; bars and error bars show the mean ± SD across three replicates. (d) Peak force, defined for each replicate as the maximum of its 1-ns-averaged force trace. Points represent replicate peak forces, and bars and error bars show the mean ± SD across three replicates. ΔSS R6877P transmits lower forces than ΔSS-KASH-2P during the early loading regime and subsequently converges toward similar force values, with overlapping replicate variability bands. M6875P shows delayed force onset relative to KASH-2P but reaches comparable forces at larger extensions. The bar-plot summaries similarly show that M6875P had a lower full-trajectory mean than KASH-2P (348 ± 13 vs 396 ± 13 pN) but a comparable peak (666 ± 68 vs 626 ± 70 pN), consistent with delayed force onset. In the disulfide-deleted background, R6877P and ΔSS-KASH-2P had comparable peaks (382 ± 38 vs 381 ± 58 pN), and their full-trajectory mean ranges overlapped (135–149 and 133–207 pN).

**Supplementary Figure 2**

Figure S2. Force propagation and point change detection of all replicate constructs. Columns show WT, ΔSS, KASH-2P, and ΔSSKASH-2P, and rows show three independent SMD replicates. For each trajectory, frames were centered and Kabsch-aligned to the centered first pulling frame before change-point detection, removing whole-complex translation and rotation from the coordinate signal. Change-point detection used an RBF kernel with γ = 0.08, a penalty of 6.3, a minimum segment size of 2, and 5,001 frames per 50-ns trajectory. Events were restricted to SUN2 residues 522–717 and retained when at least two SUN2 sites changed at the same sampled boundary. The lower plot in each panel shows the corresponding replicate-specific force trace smoothed with a centered 100-ps moving average for display. Identical tick colors within a panel denote residues assigned to the same frame-specific event group; colors do not indicate correspondence between different panels. The maps describe trajectory-specific SUN2 structural transitions and do not directly measure internal force or establish a conserved force-propagation sequence.

Figure S3. SUN2 coiled-coil RMSF as a metric of force transmission. To determine whether pulling produced a downstream structural response within SUN2, Cα root-mean-square fluctuation (RMSF) was calculated for residues 522–554 of the coiled-coil/pre-SUN region. RMSF during the final 50 ns of the production simulations (a) was compared with RMSF during the complete 50-ns pulling simulations (b). Lines represent the mean of three independent trajectories after averaging corresponding residues across the three SUN2 protomers, and shaded regions represent the standard deviation. (c) Residue-wise changes in RMSF were calculated as pulling minus production for each matched replicate and are presented as the mean ± standard deviation across the three trajectories. (d) Mean coiled-coil RMSF was calculated across residues 522–554 and the three protomers for each independent trajectory. Production and pulling values from the same replicate are connected. Constructs retaining the SUN2–KASH2 disulfide bond, WT and KASH-2P, exhibited substantially larger pulling-associated increases in coiled-coil RMSF than ΔSS and ΔSS-KASH-2P, supporting disulfide-dependent transmission of the pulling-induced structural response into SUN2.
